# MPOD: Applications of integrated multi‐omics database for medicinal plants

**DOI:** 10.1111/pbi.13769

**Published:** 2022-03-10

**Authors:** Simei He, Ling Yang, Shuang Ye, Yuan Lin, Xiaobo Li, Yina Wang, Geng Chen, Guanze Liu, Ming Zhao, Xiu Zhao, Kunhua Wei, Guanghui Zhang, Jianhua Miao, Yang Dong, Shengchao Yang

**Affiliations:** ^1^ 12616 National‐Local Joint Engineering Research Center on Gemplasm Innovation & Utilization of Chinese Medicinal Materials in Southwest China Yunnan Agricultural University Kunming China; ^2^ 12616 The Key Laboratory of Medicinal Plant Biology of Yunnan Province Yunnan Agricultural University Kunming China; ^3^ 12616 College of Food Science and Technology Yunnan Agricultural University Kunming China; ^4^ 12616 College of Tropical Crops Yunnan Agricultural University Pu'er China; ^5^ 248907 Guangxi Key Laboratory of Medicinal Resources Protection and Genetic Improvement Guangxi Botanical Garden of Medicinal Plants Nanning China; ^6^ Yunnan Plateau Characteristic Agriculture Industry Research Institute Kunming China

**Keywords:** medicinal plants, genome, transcriptome, secondary metabolite, biosynthesis

Plant natural products (PNPs) have been an important source in human nutrition, industrial raw materials, medicinal ingredients and half of anticancer drugs are derived from PNPs such as paclitaxel, vinblastine, and ginsenoside (Caputi *et al*., ; Luo *et al*., 2019; Yang *et al*., 2020). Biosynthesis is one of the key ways to produce PNPs, and the increasing development of medicinal Phyto‐omics data helps to decode the PNPs biosynthetic pathway (Liu *et al*., 2017). Genetic resources also provide the basis for medicinal plants (MPs) molecular breeding.

To integrate the genome and transcriptome data of MPs, we completed the first omics database for herbal medicine (HMOD) in December 2017 (Wang *et al*., 2018). The less genomic data and the simple metabolites information from the website, as the data increases, makes it necessary to comprehensively optimize and upgrade the database from the data, interface, tool, and management. Thus, we constructed an integrated multi‐omics database for MPs (MPOD; http://medicinalplants.ynau.edu.cn/).

MPOD collects genomes and transcriptomes of MPs published since January 2018. In addition, we sequenced six genomes, 28 transcriptomes, and five metabolomes in this study. All genomic and transcriptomic sequences in the MPOD are available for query of orthologous gene candidates, and homology comparison between gene families from different species by blast. More importantly, correlation analyses between metabolite distribution and gene expression including metabolite content in different tissues, Pearson correlation analyses of genes involved metabolic pathways and expression profile were performed. Compared with HMOD, MPOD details metabolic pathways of flavonoids, alkaloids and terpenoids, respectively. To facilitate synthetic biology, ‘the biosynthetic tools’ module is added in MPOD with some popular bioinformatics tools including SynVisio, heatmap, and enrichment.

The framework of MPOD is constructed using MySQL, ThinkPHP, and FastAdmin, with four main modules, including genomics, transcriptomics, pathways, and biosynthetic tools (Figure 1a, b). In brief, the genomics module consists of genomes, genome size, re‐sequencing, and gene (Figure 1c). This module contains 154 published genomes and 6 unpublished genome‐assemblies (*Synsepalum dulcificum, Antirrhinum majus, Platycodon grandiflorus, Codonopsis pilosula, Panax vietnamensis, Gynostemma pentaphyllum*) from this project. The web interface of species constitutes species introduction, sequencing data, assembly results, the data source links, and reference. For the published genomic data, the GCA data uploaded on NCBI has been linked to MPOD, and for unpublished data, FASTA formatted files for assembly, CDS, and protein sequences can be downloaded from this database. Genome size provides 50 plant genome size results, predicted by flow cytometry. Re‐sequencing contains single nucleotide polymorphism (SNP) information of *Erigeron breviscapus*, *P*. *notoginseng* (He *et al*., 2021) from our team, and published re‐sequencing data for 19 other plants. Gene section provides gene assembly, annotation, and expression profiles from *E. breviscapus* and *Acanthopanax senticosus*.

**Figure 1:**
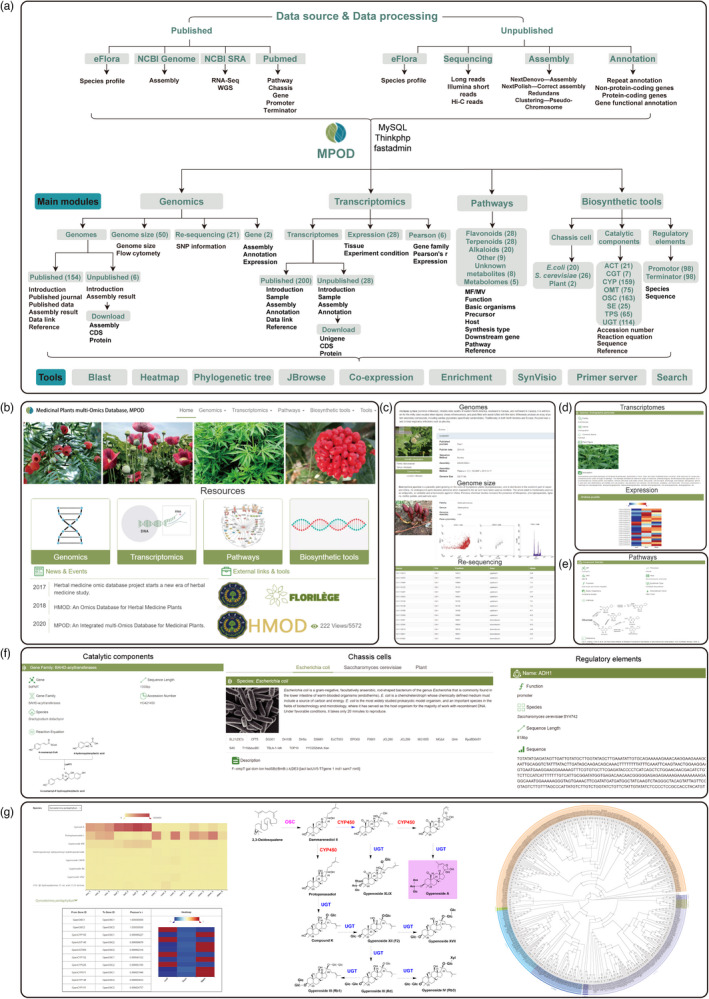
Schematic of the Database for Medicinal Plants. (a) The flow diagram showing design and construction of MPOD. (b) The home page of MPOD. (c) The ‘genomics’ module providing summary of genomes, genome size, and re‐sequencing. (d) The ‘transcriptomics’ module showing sequencing, assembly result and expression profiles. (e) The ‘pathways’ module. (f) The ‘biosynthetic tools module’ providing detailed information of catalytic components, chassis cells, and regulatory elements. (g) A case study for the application of MPOD.

The transcriptomics module contains transcriptomes, expression, and Pearson. The transcriptomes collect 200 published and 28 *de novo* sequenced data in this project (Figure 1d). It consists of species introduction, sample information, sequencing data, assembly results, annotation methods, the data source links, and reference. The transcriptome data is uploaded and linked like genomes. More importantly, for 28 unpublished transcriptomes, we provide gene expression profiles from different experimental conditions or tissues in a heatmap for easy visualization. We also perform Pearson correlation analyses of genes involved in metabolic pathways using some of our transcript expression data.

The pathways module collects 85 typical compounds whose biosynthetic pathway has been deciphered, including 28 flavonoids, 28 terpenoids, 20 alkaloids, and 9 other compounds. This module lists the compound name, molecular formula, molecular weight, function, basic organisms, precursor, host, synthesis type, downstream gene, pathway, and reference (Figure 1e). Furthermore, this module also collects 7 important compounds, but their biosynthetic pathways are not completely deciphered. Similarly, it includes type of compounds, distribution, proposed pathway, and provides the sequences and expression profiles of candidate genes potentially involved in biosynthesis. It also provides five metabolomes showing that metabolite content from different tissues using heatmap.

The biosynthetic tools module lists chassis cells, catalytic components, and regulatory elements (Figure 1f). Chassis cells present 46 strains of *Escherichia coli* and *Saccharomyces cerevisiae* commonly used in biosynthesis, and *Nicotiana benthamiana* and *Solanum lycopersicum* as a heterologous expression platform for reconstituting PNPs pathways. In the section of catalytic components, 629 enzymes from 8 major gene families that play key roles in the biosynthesis of natural products were summarized, including 21 acyltransferase (ACT), 7 C‐glycosyltransferase (CGT), 159 cytochrome P450 (CYP), 75 O‐methyltransferase (OMT), 163 oxidosqualene cyclase (OSC), 25 squalene epoxidase (SE), 65 terpene synthases (TPS), and 114 UDP‐glycosyltransferases (UGT). The accession number, gene length, sequence, reaction equation, and references are listed. The regulatory elements section presents 196 microbial promoter and terminator sequences commonly used in biosynthesis.

In addition to the main modules, MPOD provides some popular bioinformatics tools including ‘BLAST’, ‘Search’, ‘Heatmap’, and ‘JBrowse’ (Dong *et al*., 2020). All available MPOD genomes and gene models are incorporated into JBrowse. ‘SynVisio’ shows gene synteny relationships of chromosome‐level reference genomes. ‘Co‐expression analysis’ creates networks comprising sets of genes whose expressions are highly correlated.

A typical case of a user using our web is shown in Figure 1g. Gypenoside A is the main active component of *G*. *pentaphyllum*, and its content is the highest in leaves from metabolome. The biosynthesis of gypenoside A begins with 2,3‐oxidosqualene, but the key downstream enzymes OSC, CYP, and UGT have not been identified. A total of 235 CYPs from *G. pentaphyllum* (GpCYPs) were found by Blast. The phylogenetic tree was constructed based on the deduced amino acid sequences for the GpCYPs and other plant CYPs, and were distributed in eight subfamilies, namely 144 CYP71, 34 CYP85, 28 CYP72, 20 CYP86, and 4 CYP74. We also explored the expressions of GpCYPs from different tissues and presented as a heatmap. Furthermore, we performed Pearson correlation analyses of our transcript expression data among GpOSCs, GpCYPs, and GpUGTs using GpOSCs as the query gene (Figure 1g). These results facilitate the discovery of unknown genes involved in gypenoside A biosynthesis.

In summary, from genes to metabolite levels, MPOD integrates the genomics, transcriptomics, and metabolomics data of MPs published in almost recent years and sequenced in this study. These datasets provide a rich genetic resource for mining functional genes, screening molecular markers, and developing biological elements. Further combination of pathways and catalytic components greatly facilitate to decode the biosynthetic pathways of medicinal ingredients. MPOD will be continuously updated as multi‐omics data increases and new bioinformatics tools emerge, so that MPOD provides long‐term support to the research of MPs molecular‐assisted breeding and synthetic biology.

## Conflict of interest

The authors declare no conflict of interest.

## Author contributions

S. Y. and Y. D. conceived the study. S. H., L. Y., S. Y., Y. L., X. L., Y. W., G. C., G. L., M. Z., X. Z., K. W., and G. Z. collected and processed data. S. Y., Y. D., J. M., S. H., and L. Y. designed the experiments and wrote the manuscript. All the authors approved the manuscript.

## References

[pbi13769-bib-0001] Caputi, L. , Franke, J. , Farrow, S.C. , Chung, K. , Payne, R.M.E. , Nguyen, T.‐D. , Dang, T.‐T. *et al*. (2018) Missing enzymes in the biosynthesis of the anticancer drug vinblastine in Madagascar periwinkle. Science, 360, 1235–1239. 29724909 10.1126/science.aat4100

[pbi13769-bib-0002] Dong, X. , Chen, W. , Liang, Z. , Li, X. , Nick, P. , Chen, S. *et al*. (2020) VitisGDB: The Multifunctional Database for Grapevine Breeding and Genetics. Mol. Plant, 13, 1098–1100. 32416265 10.1016/j.molp.2020.05.002

[pbi13769-bib-0004] He, S. , Dong, X. , Zhang, G. , Fan, W. , Duan, S. , Shi, H. *et al*. (2021) High quality genome of *Erigeron breviscapus* provides a reference for herbal plants in Asteraceae. Mol. Ecol. Resour, 21, 153–169. 32985109 10.1111/1755-0998.13257 PMC7756436

[pbi13769-bib-0005] Liu, X. , Ding, W. and Jiang, H. (2017) Engineering microbial cell factories for the production of plant natural products: from design principles to industrial‐scale production. Microb. Cell Fact, 16, 1–9. 28724386 10.1186/s12934-017-0732-7 PMC5518134

[pbi13769-bib-0006] Luo, H. , Vong, C.T. , Chen, H. , Gao, Y. , Lyu, P. , Qiu, L. *et al*. (2019) Naturally occurring anti‐cancer compounds: shining from Chinese herbal medicine. Chin. Med., 14, 1–58. 31719837 10.1186/s13020-019-0270-9 PMC6836491

[pbi13769-bib-0007] Wang, X. , Zhang, J. , He, S. , Gao, Y. , Ma, X. , Gao, Y. *et al*. (2018) HMOD: An Omics Database for Herbal Medicine Plants. Mol. Plant, 11, 757–759. 29524650 10.1016/j.molp.2018.03.002

[pbi13769-bib-0008] Yang, Y. , Mao, J. and Tan, X. (2020) Research progress on the source, production, and anti‐cancer mechanisms of paclitaxel. Chin. J. Nat. Med, 18, 10–17. 10.1016/S1875-5364(20)60032-2 33357719

